# Improving patient‐centred care for persons with Parkinson's: Qualitative interviews with care partners about their engagement in discussions of “off” periods

**DOI:** 10.1111/hex.12884

**Published:** 2019-04-12

**Authors:** Tara Rastgardani, Melissa J. Armstrong, Connie Marras, Anna R. Gagliardi

**Affiliations:** ^1^ University Health Network Toronto Ontario Canada; ^2^ Department of Neurology University of Florida Gainesville Florida

**Keywords:** caregivers, health communication, Parkinson's disease, patient participation, patient‐centred care, qualitative research

## Abstract

**Objective:**

This study explored how care partners (CPs) of persons with Parkinson's (PwP) are engaged in discussions of “off” symptoms.

**Methods:**

During qualitative interviews, CPs of PwP sampled by convenience through the Michael J Fox Foundation online clinical trial matching service were asked to describe their familiarity with “off” symptoms, how “off” symptoms were discussed with clinicians, and the impact of “off” symptoms on them. Data were analysed using constant comparative technique by all members of the research team.

**Results:**

A total of 20 CPs were interviewed. Compared with PwP, they were more likely to describe “off” symptoms to clinicians. CPs identified important aspects of patient‐centred care for PD: establishing a therapeutic relationship, soliciting and actively listening to information about symptoms, and providing self‐management support to both PwP and CPs. CPs said that clinicians did not always engage CPs, ask about “off” symptoms or provide self‐management guidance, limiting their ability to function as caregivers.

**Conclusion:**

By not engaging and educating CPs, “off” symptoms may not be identified or addressed, leading to suboptimal medical management and quality of life for PwP. These findings must be confirmed on a broader scale through ongoing research but suggest the potential need for interventions targeted at clinicians and at CPs to promote patient‐centred care for PwP.

## 1 INTRODUCTION

Parkinson's disease (PD) is the second most common neurodegenerative disease in older adults, with annual incidence rates ranging from 8.6 to 19.0 per 100 000 population.^1^ Parkinson's disease is characterized by motor symptoms including rigidity, slowness, tremor and problems with balance that impair health‐related quality of life.^2^, ^3^ Persons with Parkinson's (PwP) also experience a wide array of non‐motor symptoms including impaired cognition, poor attention, visual disturbances, anxiety, depression, agitation, apathy, sweating, light‐headedness, urinary urgency, pain and restlessness.^3^ PD is a progressive and incurable disease so therapy goals are to relieve symptoms and preserve functional capacity.^1^ Pharmacologic treatment largely relies on dopaminergic medication; non‐pharmacologic therapies include exercise, and physical, speech and/or occupational therapy.^2^ In later disease stages, PwP who are successfully treated with medications may experience periods when motor and non‐motor symptoms worsen based on medication timing, with symptoms appearing or worsening at the end of the medication dose and then improving in response to PD therapy.^3^ Prevalence estimates for these periods, termed “off” periods, vary; as many as 25%‐50% of PwP experience symptom fluctuations as medication wears off.^3^

“Off” periods are variable in timing, severity and predominant symptoms both between and within individuals. This necessitates regular and thorough assessment by clinicians to modulate medication and provide education, which is essential to help PwP anticipate and manage motor and non‐motor symptoms, reduce potential adverse events and minimize symptom impact on quality of life.^1^ Providing PwP with self‐management knowledge, skills and confidence to monitor progress and solve problems is important to PwP.^4^ In a prior study, the majority of 492 PwP surveyed thought that self‐monitoring would improve their understanding of the condition, well‐being and ability to cope, and communication with their health‐care team.^5^ Hence, patient‐centred care (PCC), which involves communication and partnership with patients to support involvement in their own health and health care, is essential for optimal PD management.^6^ Patient‐centred care is defined as care that considers the values, beliefs, desires, lifestyle and family and social circumstances to inform, educate and engage patients consistent with their needs and values.^7^, ^8^ Patient‐centred care is considered a fundamental element of high‐quality care because it has been shown to improve patient (ie, experience, satisfaction, clinical outcomes) and health system outcomes (ie, cost‐effective service delivery and use) across multiple conditions and settings of care.^9^, ^10^ Considerable research has characterized PCC as a multidimensional approach to care that involves fostering clinician—patient relationships, involving family or caregivers/care partners, exchanging information, recognizing and responding to patient emotions, managing uncertainty, making decisions and enabling patient self‐management.^11^

Unfortunately, PCC for PD does not appear to be prevalent.In the United States, 20 Parkinson Centers of Excellence asked 50 consecutive patients to complete the patient centredness questionnaire for PD (PCQ‐PD).^12^ Significant differences between centres were found for all subscales (rated from 0 to 3); the information subscale (mean 1.62 SD 0.62) and involvement in decision‐making subscale (mean 2.03 SD 0.58) received the lowest ratings. In 30 European countries, among 1546 respondents, 34.2% said they were not involved in treatment decision making, and 17.2% said they felt rushed to make a decision.^13^ Of those, 200 were care partners (CPs), 59.0% said they helped the PwP to prepare a list of questions, and 46.5% helped to prepare a list of symptom changes prior to consultations, underscoring the role of CPs, including spouses, family members or informal caregivers, in PCC.^14^ With respect to PD, a six‐psychiatrist panel rated information provided during interviews with 223 PwP and separately by their CPs and judged that 71 (31.4%) CPs were critical for determining a psychiatric diagnosis.^15^ Without a CP, 81.3% of those with impulse control disorders and 43.8% of those with anxiety disorders would not have been diagnosed. Hence, the insight and input of CPs is essential to “off” symptom identification and control, which influences the safety, independence and quality of life of PwP, and the ability of CPs to support PwP.

Most studies on the role of CPs in PD have focused on the psychosocial, physical and financial burden on CPs of caring for PwP.^16^, ^17^ In contrast, little research has explored the role of CPs in PCC.^13^, ^15^ This is germane to the context of PD where CPs can provide clinicians with important information about “off” symptoms and symptom changes, knowledge needed by clinicians to optimize medication and symptom management. The purpose of this study was to explore how CPs of PwP are engaged by clinicians in discussions of “off” periods, including if and how CPs interact with health‐care professionals, their role in discussions about symptoms, and their potential impact on the therapeutic relationship and the care received by the PwP they represent. This knowledge may reveal whether there is a need to better engage and support CPs in PCC for PD.

## 2 METHODS

### 2.1 Approach

This study involved secondary analysis of data collected in a multi‐method study to investigate the impact on PwP and CPs of motor and non‐motor fluctuations (“off” periods) and communication about these symptoms with clinicians. Given the lack of prior research on the role of CPs in PCC for PD, the study was based on an exploratory research design involving qualitative interviews with PwP, CPs and neurologists to thoroughly identify and describe communication experiences and preferences.^18^ Interviews were conducted and analysed using a basic descriptive qualitative approach, which is neither based on nor generates theory, but instead explicitly reports experiences.^19^ In this context, it was useful to gather straightforward accounts of CP experiences and recommendations, which could provide insight on how to improve PCC for PwP.  All participants provided informed consent prior to being interviewed, and the investigators did not have any relationship with participants. To optimize rigour, we complied with all 32 items of the COREQ reporting standards for qualitative research, which includes providing details about the research team and reflexivity, study design, and analysis and reporting of the findings.^20^ To enhance rigour, we analysed responses inductively so that findings emerged from the data, compared independently derived analyses and interpretations to establish saturation and enhance trustworthiness of the findings, reviewed findings with collaborators from the Michael J Fox Foundation to further ensure credibility of the findings, and reported exemplar quotes with anonymous identifiers to illustrate the views and experiences of a variety of participants.^21^

### 2.2 Sampling and recruitment

Convenience sampling was used to recruit PwP and their CPs through the Michael J Fox Foundation community by posting a recruiting advertisement on the clinical trial matching platform Fox Trial Finder from February 13, 2017, to October 16, 2017. Fox Trial Finder is a publicly available website (https://foxtrialfinder.michaeljfox.org/) where PwP can find information about PD clinical trials and research studies and/or register to be matched with trials or other research for which they are eligible. Individuals without movement disorders can also register as controls. Since its launch in 2012, Fox Trial Finder has recruited 82 166 clinical trial/research volunteers. Interested candidates contacted a study coordinator via the Fox Trial Finder online messaging system for further information. The coordinator sent them a consent form, and once a signed consent form was returned, they were contacted to schedule an interview. Persons with Parkinson's who had experienced motor or non‐motor fluctuations who also had CPs defined as the primary carer not necessarily living with the PwP were eligible if they resided in the United States and were able to converse in English language. Sampling was concurrent with data collection and analysis. We aimed to recruit a minimum of 20 CPs and planned to recruit additional PwP and corresponding CPs if thematic saturation was not achieved. Each CP received a $100 prepaid gift card by mail in reimbursement for their time. This study focuses on CPs; findings of interviews with PwP will be reported elsewhere.

### 2.3 Data collection

All interviews were conducted by TR, a neurologist, who was trained and mentored by ARG, a PhD‐trained researcher with expertise in qualitative methods. Interviews were conducted by telephone from April 5, 2018, to October 30, 2018. Care partners were interviewed separately from PwP, and the mean duration of CP interviews was 33 minutes. Participants were asked three questions: describe your familiarity with “off” symptoms (prompts: which are most common, when and how frequently do they occur), how are you involved in communication with clinicians about “off” symptoms (prompts: attend appointments, ask questions, offer information, barriers, facilitators) and the impact of “off” symptoms on them (prompts: well‐being, relationship, usual activities). Questions were purposefully broad so that themes pertaining to their experiences would emerge from the data. As is customary in qualitative research, additional prompts were deployed if needed to elicit additional information for each question. Interviews were audio‐recorded and transcribed verbatim by a professional transcription service.

### 2.4 Data analysis

TR identified and organized themes in Microsoft Word using inductive qualitative methods with guidance from ARG.^18^, ^19^ TR read all transcripts to identify and define themes (first‐level coding). TR developed a codebook to organize themes and sample quotes. TR re‐reviewed coding (constant comparative technique) to assess whether and how to expand or merge themes (second‐level coding). Each of first‐ and second‐level coding was independently reviewed by ARG, and by MJA and CM, both neurologists with movement disorder specialization. Saturation was determined by discussion and consensus among the research team following the completion of 20 interviews. Data were reviewed by collaborators at the Michael J Fox Foundation to further ensure credibility. Data, including themes and exemplar quotes, were tabulated and summarized.

## 3 RESULTS

### 3.1 Participants

A total of 20 CPs were interviewed. They provided support to PwP with a mean age of 65.1 years (SD 8.3, range 49‐77) whose PD diagnosis was established a mean of 7.8 (SD 4.7, range 1‐20) years ago. Just over half of CPs were female (11, 55.0%). Most CPs were spouses (17, 85.0%); others were a son, brother‐in‐law and close friend.

### 3.2 Themes

Five main themes were identified: CPs familiar with “off” symptoms, CPs more likely to report and discuss “off” symptoms than PwP, CPs did not experience PCC, CPs not actively engaged in “off” discussions by clinicians, and CPs profoundly impacted by “off” periods. Overall, CPs revealed that without their input, clinicians cannot competently manage PD, potentially exposing PwP and CPs to the risk of adverse outcomes associated with poor symptom control. Individual themes with exemplar quotes are further described here.

### 3.3 CPs familiar with “off” symptoms

CPs were keenly aware of PwP “off” symptoms and associated behaviour and impact because they were involved in many aspects of the lives of PwP (Table 1). For example, they assist with administering medication, activities of daily living, monitoring for symptoms, and scheduling and planning activities. They are always with or vigilant of the PwP, while at the same time promoting some independence where possible.

We only travel together now…I'm never too far away (CP2)

**Table 1:** CPs familiar with “off” symptoms

Theme	Exemplar quote
Helps to remember and administer medication	I kind of snap to alert and I immediately check, you know, the med situation… I immediately ensure he gets them in him (CP1)
	A tremor, mostly in his right hand. Sometimes when I see that and I will ask, you know, did he remember to take his meds (CP16)
	I take emergency medicine with me in case he leaves his pills at home and we'll need them. And I always have them in my purse, so you just plan ahead (CP17)
Helps with activities of daily living	I usually offer to pick up all the slack, so I'll be cooking dinner and just take over… I just take her in stride and, you know, say, I've got this, and just kind of let her be (CP2)
	You be very attentive, you know, be there to assist the movement, try to prevent a fall especially when she's using stairs (CP10)
	There have been times I've had to cook for her (CP16)
Understands nuances of symptom timing	It's almost like he becomes a person with Asperger's; he has no eye contact, he stoops even more, he'll fix his gaze on a spot in front of him on the floor (CP1)
	We've tried really meticulously to correlate things like diet, frequency of medication, dose of medication, should she take one whole pill every two hours or a half pill every hour, that kind of stuff. We have experimented with that stuff six ways from Sunday, diet, sleep, exercise (CP2)
	She tends to feel, most of the time, very good in the morning, then at around 11 o'clock she usually has a period where she gets tired and she – if she forgets her medicine it becomes quite apparent. (CP6)
	She gets a little low on medication, then she gets some stiffness, especially with walking. It's typically for a period almost ever morning (CP9)
	She has trouble with movement, moving her feet, particularly going backwards or sideways, a little bit of trouble getting started going forward but not so much going forward (CP10)
	She's a little less patient, a little more irritable (CP14)
	The daytime one is mainly really just the shaking on the left side (CP15)
	It seems like the off periods are worse when he is fatigued, but definitely slower walking, and sometimes slight agitation, either later in the late afternoon or early evening (CP16)
	It usually entails stiffness and difficulty standing up we attribute to the medication wearing off (CP19)
Undertakes scheduling and planning of activities with and for PwP	We have had to adjust our schedules to when we do things (CP5)
	Instead of trying to do a whole bunch, we try to pace things out more (CP6)
	I need to be conscious of the time required to do things, whether it's travel or prepare a meal, and how that will impact the schedule (CP12)
Plans timing of discussions with PwP	If we need to discuss something where a decision has to be made, more than likely I'll do it in the morning, because if it's too close to the OFF time and he hasn't taken his medication and it's been in his system for maybe about an hour, I know that whatever we've talked about he either forgets or gets confused (CP5)
Is always with or vigilant of PwP	We only travel together now, she wouldn't travel alone anymore. I'm never too far away…she will frequently drive to a nearby gym or grocery store, and I'll have to pretend this is normal, but also double‐checking that she's okay to drive right now (CP2)
Also attempts to support some independence	But whenever possible I want to have him do the kind of decision making that he can handle (CP15)
	The independence thing, you know. She wants to feel like she's needed and I don't want to take away any of her independence until I absolutely have to (CP16)
	A coping strategy for me is not to hover. If he's doing something and it's taking longer than it should, I look at him and say ‘I'm gonna go in the other room right now and if you need my help, just let me know' (CP18)

CPs, care partners; PwP, persons with Parkinson's.

Care partners spoke about recognizing symptoms such as irritability or lack of attention, and carefully timing discussions and activities with PwP to accommodate “off” periods.

I need to be conscious of the time required to do things (CP12)

### 3.4 CPs more likely to report and discuss “off” symptoms than PwP

Care partners said they prepared for and accompanied PwP to consultations (Table 2). In particular, CPs said they were more likely to report “off” symptoms to physicians than PwP, who are not aware of, or may deny or hide the extent of their symptoms.

He won't accept “off” symptoms. I tend to be more upfront and let her [the doctor] know what's going on, where he's more let's see if it gets worse, we don't need to tell the doctor right now (CP5)

He's not a good communicator with his doctor as far as his symptoms. He kind of forgets when he's had a bad symptom (CP17)

**Table 2:** CPs more likely to report and discuss “off” symptoms than PwP

Theme	Exemplar quote
Prepares for appointments	I have about 8 weeks of charts that I'm going to take. I have taken a paper and put a grid on it. I'll take that in and show him (CP2)
	We both will make notes if something unusual happens and keep a list of those things and then go over them with her so that we're prepared (CP5)
	I do a written preparation for every doctor that we, I mean even the dentist. I take a list of all the meds because, you know, he's taking a lot (CP15)
Attends all appointments	I go to all doctors' appointments with him, every one of them, because he doesn't remember things, or he'll … not intentionally, but he'll forget to tell things (CP5)
	I go to all the appointments with her, you know (CP6)
	I'm there when she has an appointment (CP10)
Remembers or is aware of symptoms and details	I'm in on every doctor's appointment that he has because I want another set of ears listening to what's being said, cause a lot of times she doesn't remember (CP3)
	She likes it when I go with her and I don't mind doing that because it gives another set of perspectives and memory of what might have happened (CP7)
	Doctor's name asked her yesterday about what percentage of the day did she have dyskinesia. And wife's name said, “Well, not much. Maybe 10%.” I had to say, “That's wrong.” I wish it was, but I would say minimally 40%, 50% of the day (CP8)
	He's not a good communicator with his doctor as far as his symptoms. He kind of forgets when he's had a bad symptom (CP17)
Prompts/raises discussion of symptoms	They question me a lot as to try to discover how he's doing, how the symptoms are doing and should there be any medicine adjustments. I tend to bring up the cognitive problems…they don't seem particularly interested in dealing with it (CP1)
	I need to make a list of questions. I usually go in there with quite a list and quite a stack of documents (CP2)
Provides more complete or accurate information than PwP	He works extra hard when we're at the doctor to walk up straight and all of this. I tend to bring up more of his problems than he does (CP4)
	He won't accept “off” symptoms. I tend to be more upfront and let her [the doctor] know what's going on, where he's more let's see if it gets worse, we don't need to tell the doctor right now (CP5)
	She and I were sitting side‐by‐side and the doctor was across the room. And when she said 10% I put my thumb pointing up and indicated it was more than that. I don't think she's even aware of it sometimes but I'm sitting on the other side watching and it's a lot more than that. And [my wife] didn't disagree. She said, “Well, you know, you're right. It just gets to be part of the day and I don't think about it.”
Absorb information given by physician	We've learned that for either of us it's necessary for the partner to be in the room with the doctor because you don't hear, especially when there's bad news (CP8)
	I always go with him. He takes his whole folder of information; I take my notes. He and I both believe that four ears are better than two (CP18)
Monitors for new research findings on PD	We belong to a local Parkinson's group that meets monthly so we get a lot of information there and I'm usually online looking to see if anything new is coming up (CP5)
	We're pretty Internet literate so we go on the Internet and find sources on there (CP9)

CPs, care partners; PD, Parkinson's disease; PwP, persons with Parkinson's.

### 3.5 CPs did not experience PCC

Care partners reported variable PCC practices among physicians. Those who described good communication practices said that physicians made general enquiries about family and activities before launching into medical issues, took time to listen, conveyed interest, maintained eye contact and did not simultaneously enter data into a computer, helped to find solutions, and acknowledged that everyone is different and did not dismiss concerns.

He spends all the time in the world with us and does listen to what we are saying (CP3)

She actually sits and listens, and she doesn't try to make you think that something else is happening, you know like she doesn't say well, no, that can't be…because everybody is different and some things affect people in different ways, so she never says, well, you know, that's not possible (CP5)

However, several CPs reported suboptimal PCC (Table 3). Those CPs said that physicians did not convey empathy, did not actively prompt for information about symptoms, ignored cognitive issues when raised, and focused attention on their computer. Care partners also thought that appointments for monitoring “off” symptoms should be more frequent.

The doctor is forced to sit down and fill out little frames on the computer and not really sit down and talk with the patient to get an idea of what's going on in their life, you know, what used to be a holistic approach to medicine (CP6)

I think she's a good doctor but she said something the other day that sure bothered me: “You're not the only person in the world with Parkinson's, you know?” It really pissed me off (CP8)

**Table 3:** CPs did not experience PCC

Theme	Exemplar quote
Lack of individual approach or empathy	They're not going to treat her, they're just going to follow her. And that's what it feels like, they're like following, but it doesn't feel like an advocacy for us as we kind of thought they would be more interested or something (CP2)
	I think she's a good doctor but she said something the other day that sure bothered me: “You're not the only person in the world with Parkinson's, you know?”. It really pissed me off (CP8)
Ignore symptoms or issues considered important by CP	They have no skill for his cognitive issues and they don't seem particularly interested in dealing with it (CP1)
Do not actively prompt for information about symptoms or issues	It certainly would help for the physician to ask open‐ended questions and be patient to listen to the responses (CP1)
	Her doctors have been – aloof isn't quite the right word – but we only them once every six to nine months, and they're not too keen on getting the scoop…they're certainly not asking the level of questions, like what is the impact and extent and severity of these “off” periods (CP2)
Focus on computer system rather than communication	I feel that he's very distracted by the technology that they use, those computers. And I feel like he has his checklist, and he really does need to get through that before he can really focus on us (CP2)
	If you don't have that [communication with PD patient and CP] it's much more difficult to treat the patient well…It's become tougher with changes in medicine to maintain good communication…a lot of time the doctor is forced to sit down and fill out little frames on the computer and not really sit down and talk with the patient to get an idea of what's going on in their life, you know, what used to be a holistic approach to medicine (CP6)
Infrequency of monitoring not sufficient	I think maybe more frequent communication would be good, but the doctor at this point doesn't think it's necessary (CP3)
	I think the neurologist is really, really good, but by definition that means he's really, really busy and I probably would like more frequent communication whereas it can be six to eight months between visits (CP7)

CPs, care partners; PCC, patient‐centred care.

### 3.6 CPs not actively engaged in “off” discussions by clinicians

Care partners that described good communication also noted that physicians were open to talking to both the PwP and CP. In contrast, several CPs described suboptimal CP engagement in PCC. Some physicians did not give or receive information to/from CPs:

I think the neurologist should enquire a whole lot more of me. I mean, they obviously need to direct themselves towards my husband as a person with Parkinson's and all of that kind of thing, but I think they miss and have missed a whole bunch of data and information, and it took me a couple of years, not because I'm not assertive or anything, but it was kind of a new experience to be my husband's spokesperson, but I used to sit there and think they aren't getting half the story just listening to how my husband perceives what's happening with him, and I thought it was really odd that the reaction to the person who is with him 24/7 isn't more important to these neurologists (CP1)

They've never turned to me and asked, so if I've told them anything it's only because I felt the urge to chirp up and say something…they don't seem to get that you are the only authoritative person who can communicate what these “off” periods are like and how frequent they are and how unpredictable they are. It's been a struggle; as somewhat introverted, I probably don't chip in as much as I should (CP2)

Care partners also said that physicians did not provide them with guidance on how to provide care to PwP:

Caregivers need training, we need support, we need all kinds of things, and not just told to take ourselves and get some respite…It took me a long time to learn some practical things, like how to help my husband out of a chair so it would not wreck my back (CP1)

It would be tremendously important because we don't know what to do. I've asked more than once and I can't really think I've got a clear answer on what I should do, should I push her to keep moving or should I force her to sit down until it passes (CP2)

### 3.7 CPs profoundly impacted by “off” periods

Care partners described numerous detrimental sequelae and profound impacts associated with their roles as caregivers and as care partners (Table 4). These included anxiety, stress, helplessness, distress, uncertainty about the future, frustration, sadness and fear. “Off” symptoms also limited CP personal and professional activity, and limited joint activities of PwP and CPs.

Helplessness, like you can't do anything to help (CP4)

I can see what I think is fear…and that makes me feel anxious because she's not in control (CP7)

He felt less able to do things which made us both feel less able to do things (CP12)

**Table 4:** CPs profoundly impacted by “off” periods

Theme	Exemplar quote
Anxiety	I can see what I think is fear…and that makes me feel anxious because she's not in control (CP7)
	I certainly get anxious concerning a fall (CP10)
Stress	[referring to OFF periods] It's an added weight about dealing with him and with his situation, so yeah it impacts my life, it adds to the stress…if it was more predictable I would probably get myself really prepared…because it's so unpredictable I think it's more stressful (CP1)
	And all of a sudden he's calling me to help him get up. He fell in the bedroom and just missed hitting his head on the corner of the dresser. So it's very, very stressful for me (CP3)
Helplessness	Helplessness, like you can't do anything to help (CP4)
	I'm a fixer and I can't fix it (CP7)
Uncertainty	He's clearly on the road to dementia and I've a lot of questions about what's ahead for us, how bad will that get and so on (CP1)
	That's the thing, I mean with Parkinson's and everybody's so individual it's the unknown that really gets me scares (CP5)
Distress	They're absolutely awful for me [referring to “off” periods]. I hate watching her figuratively go downhill (CP16)
Frustration	The thing that's so frustrating about being a care giver is dealing with so many different symptoms (CP1)
	Instead of just sitting down or taking measures to ease the OFF period, that's where the stubbornness comes in and that's why I get frustrated, if he would just sit down and rest for a few minutes (CP5)
	It's frustrating to see her because there's really nothing I can do to help her (CP9)
	I think there was a lot of frustration and I would find myself getting short tempered over certain things like, “Why aren't you remembering this, why are you doing that sort of thing?” (CP12)
	It's frustrating when you really cannot do a whole lot, where she is dependent upon certain physical activities and medication to try to snap out of the “off” time (CP13)
Sadness	I feel very sorry for him to tell you the truth (CP17)
Fear	I feel horrid, I really do. I'm afraid that he's going to hurt himself, depending on the freezing position that he's in. I worry about him falling, which he has done a few times (CP3)
	It's like her feet don't respond anymore and she went head first into a rock and severely cut her forehead. We were out in the middle of nowhere. I was terrified. I've always been pretty good when there's an emergency but I was absolutely paralyzed, I didn't know what to do (CP18)
Reduced couple activities	It's hard to plan anything in the evening anymore because that seems to be his worst time (CP3)
	We won't go on a trip where we have to do a whole bunch of hiking. The levels of our activities have changed (CP6)
	She wants to travel and we can't do that. We haven't been able to do much of anything socially for the last couple of years because of this. It had a pretty severe effect on our social lives and her ability to function in a wider society. That really is limiting (CP7)
	If it has to do with crowds, like going to a fair or going to a shopping mall or going to a concert venue, we won't do (CP9)
	He felt less able to do things which made us both feel less able to do things (CP12)
	We don't go to the movies. We don't go to the theatre. We don't go to the Philharmonic, which was a favourite of ours (CP15)
Reduced individual activities	I was doing a fair amount of traveling and that has been curtailed because it's not good to leave her (CP2)
	I have a lot of friends that I like to go to lunch and shopping and things with, and it's hard because all I do is worry about him because he's alone (CP3)
	I find myself not doing as many things as I used to (CP5)
Impact on career	There's no way I could consider a fulltime position right now…so I work from home and I take coaching calls…I was doing a fair amount of traveling but that has been curtailed because it's not good to leave her (CP2)
	Sometimes I'll have to go to work late. I'll have to call in and have to deal with that [referring to symptoms] (CP16)

CPs, care partners.

## 4 DISCUSSION

Care partners hold dual roles as caregivers and care partners for PwP; they support activities of daily living, and they prepare for and accompany PwP to appointments. As a result, they are aware of “off” symptoms. Care partners are also more likely to communicate symptoms to clinicians compared with PwP. Care partners of PwP emphasized important components of PCC for PD that were often lacking including: make general personal enquires to establish the basis of a therapeutic relationship, better understand the impact of “off” periods on daily life, solicit and convey interest in information about symptoms and other concerns, and take time to listen and only then enter data into a computer system, and interact with, and provide education and self‐management support to both PwP and CPs. Given that not all clinicians actively prompted PwP or CPs for information about “off,” important details about symptoms may be missed. Without CP input, clinicians cannot competently manage PD, potentially exposing PwP, CPs and clinicians to the risk of adverse outcomes associated with poor symptom control. Furthermore, most CPs were not engaged in PCC by clinicians or provided with education that would help the CP to better care for PD patients. By not engaging CPs, the many detrimental ways that CPs are affected by “off” periods may go unrecognized, limiting the clinician's ability to appropriately counsel and support the CP in their caregiving role to the PwP. Overall, these findings suggest that, by not engaging CPs, PCC for PD may not be taking place, which results in a suboptimal care experience. It may also compromise medical care for PwP if “off” symptoms are not revealed and addressed, reducing health‐related quality of life, and placing additional burden on CPs who are not optimally engaged in, supported and prepared to care for PwP.

Our study empirically supports the findings of research on CP roles in general. Commentaries not related to PD have identified the emotional, physical and psychosocial demands on CPs, and the unique insight of CPs on symptoms and treatment effect.^22^ They have also advocated for the critical need to integrate CPs in PCC by: ensuring they, along with the patient, understand the patient's underlying health status; eliciting and prioritizing goals of care from CPs of patients who cannot advocate for themselves; involving CPs in establishing a care plan; and offering opportunities for CPs to individually consult with members of the health‐care team.^23^, ^24^ A systematic review of triadic medical consultations reported that companions desired involvement in decision making but felt they were actively excluded.^25^ Field observation of 30 older patient—family companion dyads during primary care visits found that more than half of companion communication behaviours were directed at improving physician understanding of the patient.^26^ Thus, while our research was specific to the context of PD, CPs in general should be better engaged by clinicians.

Our qualitative findings based on the views and experiences of CPs of PwP also align with the only prior study on the role of CPs in PCC, which was based on the opinion of psychiatrists.^15^ Moreover, our study is unique from previous research because it also revealed that CPs of PwP may not be consistently engaged in PCC for PD, an important insight on how to potentially improve communication about “off.” Interviews with 26 clinicians in the United Kingdom about PCC for persons with long‐term conditions including PD revealed tensions and uncertainties in how to balance professional judgement with patient preferences and circumstances.^27^ This suggests that clinicians may require training and support to deliver PCC, and also to understand how to best engage CPs of PwP in PCC. Thus far, efforts to improve PCC for PD have targeted patients through educational meetings and material, support groups or decision aids.^28^ Thus, strategies to train and influence clinicians must be implemented. A Cochrane systematic review showed that PCC is more probable if strategies to support it are aimed not only at patients or CPs, but also at physicians who influence treatment choices.^29^ Others have noted that teaching of communication skills often receives little attention during medical training, and little to no attention after medical training, and recommended continuing education programmes employing not only lectures, but also role‐playing, coaching and feedback from standardized patients to impart skill in PCC.^30^

Clinician education or training alone may be a simplistic and insufficient intervention given insight from CPs that highlighted long wait times between appointments. This may suggest that access to care for PwP and the lack of time with PwP and CPs may be influenced by long wait lists, which in turn could reflect a lack of movement disorder specialists and/or lack of other specialists such as nursing, psychiatrists, neuropsychologists, speech or language therapists and physiotherapists to provide multidisciplinary care. Many CPs in this study implied they had access to movement disorders specialists, but many individuals in the United States and elsewhere may not have access to subspecialty care. Telemedicine and comprehensive care clinics are potential ways to improve access to specialists, but evaluations have shown that their reach and services are limited.^31^, ^32^, ^33^ Hence, further research is needed to understand how to overcome system‐level barriers of access to high‐quality PCC for PwP.

Recent reviews highlighted the burden of PD on CPs in their caregiving role.^16^, ^17^ Our study also identified the profound impact of caregiving that results in anxiety, stress, helplessness, distress, frustration, sadness and fear among CPs of PwP. Our study also revealed the confusion and concern experienced by CPs in consultations when they were uncertain about whether and how to contribute while simultaneously being alarmed that key details that only they could contribute were being missed. This underscores the need to provide CPs of PwP with support for their role as care partners in health‐care planning. Numerous instruments are available to assess caregiver burden,^17^ but not to assess CP interest and capacity to be involved in PCC for PD.^34^, ^35^, ^36^ Thus, further research is needed to specifically investigate how to best engage CPs of PwP in health‐care planning, and the best type of intervention to support CPs.

Strengths of this study included the use of rigorous methods for data collection and analysis including independent review by multiple authors to establish and interpret themes, sampling to thematic saturation and compliance with standards for the conduct and reporting of qualitative research.^20^, ^21^ Still, the interpretation and application of these findings may be limited by several issues. Sampling was convenience rather than purposive, and we did not collect data on race/ethnicity, education or stage of disease, so we were not able to explore possible differences in views and experiences among PwP and CPs that varied in these characteristics. A small number of PwP and CPs were interviewed; thus, their representativeness of the larger population of PwP and CPs is unclear. We did not ask participants to review the results (member‐checking) and confirm our interpretation. However, the purpose of qualitative research is to identify issues and their cause to hypothesize possible relationships and/or solutions. Participants resided in the United States so the findings may not be transferrable to other settings or types of health‐care systems.

Despite the potential limitations, the purpose of qualitative research is to reveal concepts that may warrant ongoing investigation. Future research could verify these findings by surveying larger numbers of PwP and CPs as well as clinicians in the United States and elsewhere. Further qualitative research may be needed to understand how to balance the role of CPs so that PwP needs and preferences are respected. Clearly, research is needed to develop interventions for PwP, CPs and clinicians that optimize PCC and the involvement of CPs in PCC for PD, and how to implement those interventions. That research could also establish the association between interventions and desirable outcomes including but not limited to the PwP and CP experience of care, the extent of PCC, and the impact on PwP and CP clinical and psychosocial outcomes, and on clinician confidence and satisfaction in managing PD.

## 5 CONCLUSIONS

Patient‐centred care is an approach that tailors care to patient clinical needs, life circumstances and preferences. An important component of PCC is CP engagement. This study confirmed the critical role of CPs of PwP in accurately and thoroughly communicating “off” symptoms to clinicians, knowledge that is required to optimize medication type and dose, and quality of life. CPs we interviewed identified important aspects of PCC for PD including establishing a therapeutic relationship with and engaging both the PwP and CP to solicit information and actively listen to information about symptoms and other concerns, and provide education and self‐management support. CPs said that clinicians did not always ask about or address symptoms when raised, and did not always engage CPs in discussions. As a result, important information about “off” symptoms was not considered, and CPs were not provided with strategies for dealing with “off” symptoms, further adding to CP burden and potentially resulting in suboptimal management of PD. As a qualitative study, these findings must be confirmed on a broader scale through ongoing research. However, the findings suggest the need for interventions targeted at clinicians and at CPs to promote PCC for PD.

## CONFLICT OF INTEREST

The authors declare no conflicts of interest.
